# Incremental value of coronary computed tomography angiography in addition to invasive coronary angiography in MINOCA

**DOI:** 10.1007/s10554-025-03401-1

**Published:** 2025-04-21

**Authors:** Oscar Winnberg, Elin Brolin, Shams Y-Hassan, Loghman Henareh, Peder Sörensson, Olov Collste, Christina Ekenbäck, Magnus Lundin, Kenneth Caidahl, Stefan Agewall, Kerstin Cederlund, Jannike Nickander, Martin G. Sundqvist, Claes Hofman-Bang, Patrik Lyngå, Eva Maret, Nondita Sarkar, Jonas Spaak, Rehana Parvin Roshnee, Martin Ugander, Irene Santos-Pardo, Per Tornvall, Jens Jensen

**Affiliations:** 1 Department of Clinical Science and Education, Södersjukhuset, Karolinska Institutet, and Cardiology Unit, Sjukhusbacken 10, 118 83 Södersjukhuset, Stockholm, Sweden; 2 Department of Clinical Science, Intervention and Technology, Karolinska Institutet, Stockholm, Sweden; 3 Department of Radiology, Capio S:T Görans Hospital, Stockholm, Sweden; 4 Department of Cardiology, Karolinska University Hospital, Karolinska Institutet, Stockholm, Sweden; 5 Department of Medicine Solna, Karolinska Institutet, and Coronary Artery Disease Area, Heart and Vascular Theme, Karolinska University Hospital, Stockholm, Sweden; 6 Cardiology Unit, Södersjukhuset, Stockholm, Sweden; 7 Department of Clinical Sciences, Division of Cardiovascular Medicine, Karolinska Institutet, Danderyd Hospital, Stockholm, Sweden; 8 Department of Molecular Medicine and Surgery, Karolinska Institutet, and Department of Clinical Physiology, Karolinska University Hospital, Stockholm, Sweden; 9 Institute of Clinical Medicine, University of Oslo, Oslo, Norway; 10 Department of Radiology, Södertälje Hospital, Södertälje, Sweden; 11 Department of Medicine Solna, Karolinska Institutet, Stockholm, Sweden; 12 Faculty of Medicine and Health, Kolling Institute, Royal North Shore Hospital, and Charles Perkins Centre, University of Sydney, Sydney, Australia; 13 Department of Clinical Science and Education, Södersjukhuset, Karolinska Institutet, and Department of Cardiology, Capio S:T Görans Hospital, Stockholm, Sweden; 14 Department of Global Public Health, Karolinska Institutet, Stockholm, Sweden; 15 Department of Clinical Science and Education, Södersjukhuset, Karolinska Institutet and Unit of Cardiovascular Interventions, Heart Institute, Germans Trias I Pujol University Hospital, Badalona, Spain

**Keywords:** MINOCA, Atherosclerosis, CCTA, ICA

## Abstract

Patients with the working diagnosis myocardial infarction with nonobstructive coronary arteries (MINOCA) have diverse underlying causes warranting further investigations. Despite the documented superiority of coronary computed tomography angiography (CCTA) over invasive coronary angiography (ICA) in plaque detection, the former is not routinely recommended for MINOCA patients, highlighting a knowledge gap regarding CCTA’s incremental value. The objective of this study is to assess the prevalence and extent of coronary atherosclerosis in MINOCA patients using CCTA, and to evaluate the incremental value of CCTA over ICA alone in detecting coronary atherosclerosis. The data from 163 MINOCA patients who underwent both CCTA and ICA in two prospective studies were retrospectively analyzed to compare the occurrence and distribution of coronary atherosclerotic plaques detected with ICA versus CCTA, evaluating CCTA’s incremental value. CCTA detected coronary atherosclerosis in 48% of subjects; ICA did so in 47%. Notable disagreement, reflected by kappa values of 0.34 (95% confidence interval [CI] 0.19–0.48) across all segments and 0.41 (95% CI 0.27–0.55) for proximal segments (both *p* < *0.0001),* highlighted discrepancies between CCTA and ICA in the detection of atherosclerosis presence and location. Combining CCTA with ICA provided significant incremental value in detecting atherosclerosis in coronary segments (*p* < *0.001*). MINOCA patients frequently exhibit non-obstructive coronary plaques. Agreement between CCTA and ICA is poor. CCTA provides valuable additional information on atherosclerotic segments. Therefore, CCTA should be recognized as a complementary tool to ICA, aiding risk assessment and treatment decisions in the context of MINOCA.

**Supplementary Information:**

The online version contains supplementary material available at 10.1007/s10554-025-03401-1.

## Introduction

Myocardial infarction with nonobstructive coronary arteries (MINOCA) is common and accounts for 6–9% of all myocardial infarctions (MIs) [1, 2]. The underlying causes of MINOCA are heterogeneous and can be divided into coronary, cardiac, or non-cardiac [3]. Coronary causes include plaque disruption with subsequent thrombus formation, coronary embolism, intimal dissection, and vasospasm. Cardiac causes include arrhythmia, myocarditis and takotsubo syndrome. MINOCA should be viewed as a “working diagnosis” and further investigation of the underlying cause should be pursued [4]. Multimodal imaging is recommended, including prompt cardiovascular magnetic resonance imaging (CMR) and invasive diagnostic modalities, such as intravascular ultrasound or coronary optical coherence tomography, in addition to the default invasive coronary angiography (ICA) [4–6].

Non-invasive coronary computed tomography angiography (CCTA) has been shown to be superior to ICA in detecting atherosclerotic disease [7, 8], but is not recommended in the recent European Society of Cardiology guidelines for the management of acute coronary syndromes [9]. Previous studies where MINOCA patients were examined with ICA and CCTA showed that CCTA found more atherosclerotic plaques than ICA [10–12]. These studies were limited by low numbers of participants (25–50 patients), leaving a knowledge gap regarding the incremental value of CCTA in detecting coronary atherosclerosis in MINOCA.

The first aim of this study was to investigate the prevalence and extent of coronary atherosclerosis in MINOCA patients using CCTA. The second aim was to compare CCTA findings with those of ICA to determine if there is any incremental value of CCTA in detecting coronary atherosclerosis in MINOCA.

## Methods

### Study design

This is a sub study based on data collected from the Stockholm myocardial infarction with normal coronaries studies, SMINC-1 [13] and SMINC-2 [3]. These were prospective, non-randomized, multicenter studies conducted in Stockholm from 2007 to 2012 (SMINC-1) and 2014 to 2018 (SMINC-2). The diagnostic criteria for MINOCA was MI as described in the third universal definition of MI [14], with ICA showing no lesion with a diameter stenosis exceeding 30 and 50 percent of the artery lumen in SMINC-1 and SMINC-2, respectively. Subjects aged 35–69 years with a sinus rhythm on the admission electrocardiogram (ECG) were included, and examined with ICA, CMR, echocardiography and CCTA. Exclusion criteria were previous MI, pulmonary embolism, known cardiomyopathy, severe chronic obstructive pulmonary disease, and renal impairment defined as s-creatinine > 150 µmol/l.

Subjects who could not participate due to claustrophobia or had a cardiac device (pacemaker or implantable cardioversion device) were excluded. The studies were carried out in compliance with the Declaration of Helsinki and good clinical practice. Ethical approval was obtained from the Stockholm Regional Board of Ethics (2007/1583-32, 2009/1966–322014/131-31/1, 2014/131-31/1, 2014/1546-32) and all subjects provided written, informed consent.

### Study group

A total of 100 patients were included in the SMINC-1 study and examined with ICA and CMR, with 61 of them also examined with CCTA as described by Brolin et al. [15]. Four subjects in SMINC-1, whose CCTA images were considered non-evaluable or who had less than seven assessable segments, were excluded, leaving 57 subjects from SMINC-1. The SMINC-2 study included 150 patients, of whom 131 were examined with ICA, CMR and CCTA. No subject from SMINC-2 was excluded due to non-evaluable CCTA images. The CCTA was performed 3–6 months (SMINC-1) or 1 month (SMINC-2) after the acute event. Dropouts from SMINC-1 and 2, four subjects in total, were due to atrial fibrillation, claustrophobia, previous adverse reaction to iodine-based contrast agent, and logistical reasons as described by Sorensen et al. [3] and Brolin et al. [15]*.* In this retrospective analysis, all patients presenting with signs of myocarditis on CMR imaging (n = 25) were excluded, leaving a total of 163 subjects available for analysis*.* While TTS is no longer considered a cause of MINOCA, it remains an important differential diagnosis in patients initially suspected of having MINOCA. At the time SMINC-1 and SMINC-2 were conducted, TTS was classified as part of MINOCA, and therefore, patients with TTS were included.

### CCTA data acquisition and analysis

CCTA data acquisition is described in more detail in the Supplementary Materials. The CCTA examinations were independently analyzed by two experienced readers (American College of Cardiology Foundation/American Heart Association level 2 [16]) who were blinded to all clinical information. Joint readings were subsequently performed to reach consensus. CCTA data analysis was performed using either the CardIQ Xpress software on the Advantage Workstation 4.4 (GE Healthcare, Milwaukee, Wisconsin, USA) or the syngo.via software on a PACS workstation (Siemens Medical Solutions, Forchheim, Germany). Axial source images and multiplanar and curved multiplanar reformats were used. The optimal image display settings for lumen and plaque assessment were chosen on an individual basis (in general at a window width of 800–1000 Hounsfield units (HU) and a level of 100–200 HU). Coronary arteries were subdivided into 17 segments, in accordance with the modified American Heart Association classification [17]. Each segment was first assessed regarding image quality and evaluability. Segments were considered non-evaluable if artifacts prevented reliable assessment of the lumen or the vessel wall due to motion or image noise. Then, each segment was visually evaluated for the presence of atherosclerotic plaques, defined as any structure, discernible in at least two planes, within or adjacent to the vessel lumen, which could be clearly separated from the vessel lumen and from adjacent soft tissue. Lesions were quantified in regard to stenosis through visual estimation; this was expressed in terms of diameter stenosis: < 20%, 20–50% or ≥ 50%.

Plaque composition was visually assessed based on the presence or absence of calcified tissue, categorized as non-calcified, partially calcified, or calcified (with > 50% calcified components). Further assessment of high-risk plaque characteristics was not performed. The coronary calcium score was reported in terms of Agatston Units, based on the Agatston scoring algorithm [18]. The calcium score was calculated using semi-automatic software, either the SmartScore 4.0 (GE Healthcare, Milwaukee, Wisconsin, USA) or the syngo.via software (Siemens Medical Solutions, Forchheim, Germany). More detailed information can be found in the supplementary materials.

### Invasive coronary angiography (ICA)

All subjects underwent ICA at the time of initial hospital admission and the examinations were performed in accordance with local clinical practice at the participating hospitals. Six to eight projections of the coronary arteries were made. In some cases, the examination was supplemented with left ventricular angiography. Only a visual assessment was made. No intracoronary flow measurements or other imaging techniques were used.

### ICA analysis

The images were reviewed independently by two experienced interventional cardiologists who were blinded to all clinical data. The coronary arteries were assessed at the segment level using the modified American Heart Association 17 segment classification [17].

Lesions were quantified for stenosis or plaques through visual estimation, comparing the minimal lumen of the stenotic segment with the lumen of the adjacent proximal unaffected segment, and classified as no plaque, plaque with diameter stenosis < 50% or ≥ 50%, or occlusion. In the event of any discrepancy in the assessments, agreement was reached by consensus decision.

### Statistics

Values for continuous variables are reported as medians with ranges. Values for dichotomous variables are reported as counts with percentages. We dichotomized the interpretation data from CCTA and ICA; coronary arteries with no segments with atherosclerosis were considered normal and coronary arteries with one or several segments with atherosclerosis were considered pathological. McNemar’s test was used for comparison of dichotomous variables between the two methods. The incremental value of CCTA was considered to be the difference between interpretations based on ICA alone and interpretations based on ICA and CCTA combined. Cohen’s kappa coefficient [19] was used to measure inter-method agreement between CCTA and ICA regarding normal or pathological coronary arteries. A kappa value of 0.21–0.40 indicates fair agreement, 0.41–0.60 indicates moderate agreement, and 0.61–0.80 indicates substantial agreement [20]. Statistical analyses were performed using Microsoft Excel® (Microsoft, Redmond, Washington, USA) and IBM SPSS Statistics® (IBM SPSS Statistics 26, Armonk, New York, USA). A *p*-value < 0.05 was considered to be statistically significant. When appropriate, 95% confidence intervals (CI) are presented.

## Results

**Fig. 1:**
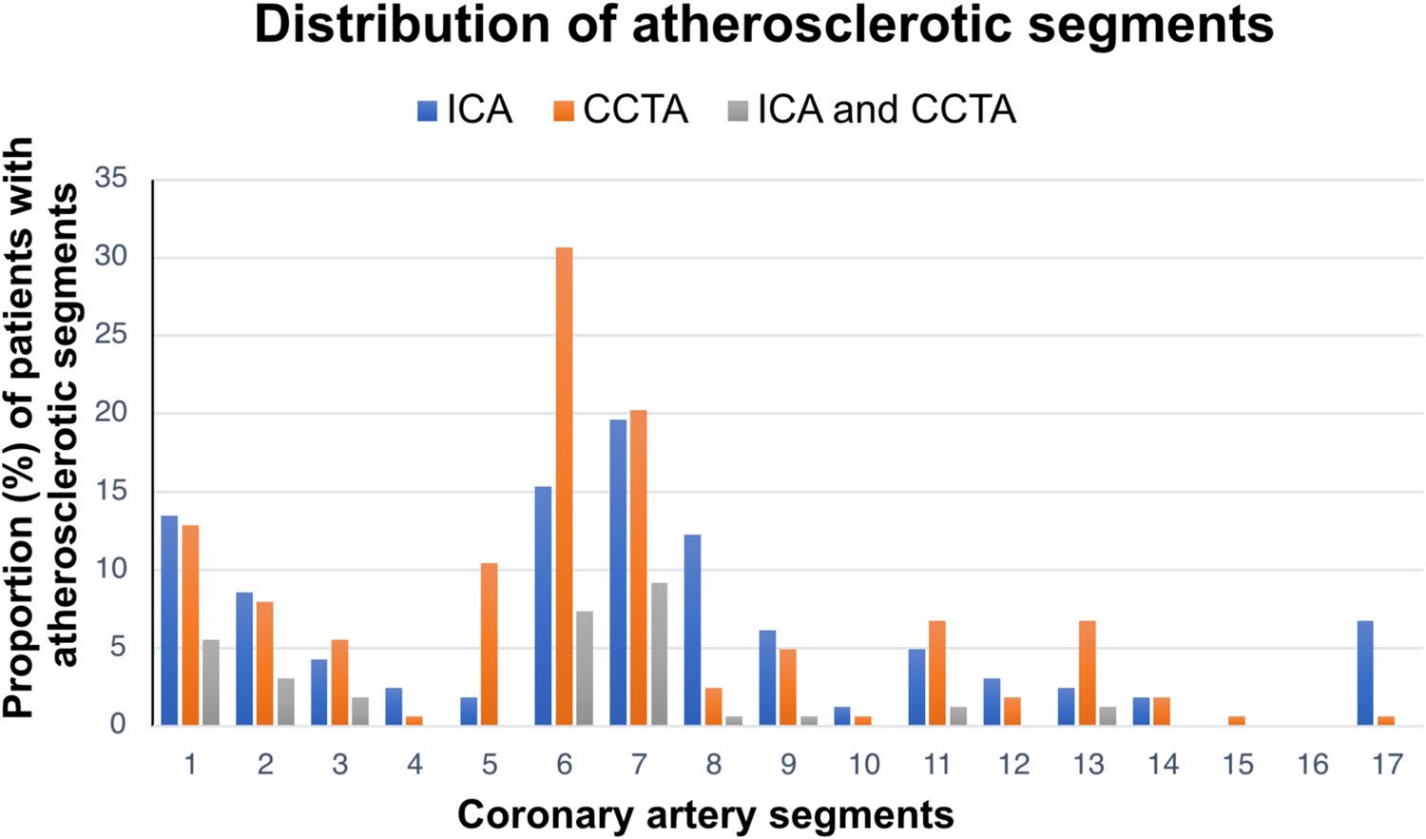
Distribution of atherosclerotic segments. Atherosclerotic segments seen with ICA and CCTA separately and with both methods (agreement) at a patient level (proportions). The number of segments is shown as a percentage. *CCTA* coronary computed tomography angiography, *ICA* Invasive coronary angiography

Baseline characteristics are shown in Table 1. The subjects’ mean age was 59 years, and 74% were female. CMR findings are categorized and presented according to diagnoses. The distribution of coronary atherosclerotic segments is presented in Fig. 1. Normal coronary arteries were found in 52% of subjects with CCTA and in 53% of subjects with ICA. In most cases where atherosclerosis was present, only a few segments were involved. CCTA found no stenoses with a diameter > 50% (diameter stenosis). The proximal left descending artery (segment 6) was the one most often affected. Nonobstructive calcified plaques were observed in 39% of the subjects, while mixed plaques were found in 13%, and non-calcified plaques in 8%. The median coronary artery calcium (CAC) score for patients with any detectable calcium was 24 AU. For those with a normal ICA, the median CAC was 15 AU, whereas for patients with atherosclerosis on ICA, it was 47 AU. A CAC > 100 was observed in 8.6% of patients, while 3.1% had a CAC > 300. Details on CCTA plaque burden and composition are provided in Supplemental Table 1.

**Table 1:** Baseline Characteristics of study participants

	MINOCA patients (n = 163)
Age, yrs	59 (36–69)
Female	121 (74)
Current smoker	29 (18)
Family history of CAD	47 (29)
Diabetes mellitus	8 (5)
Treated hypertension	38 (23)
Treated hyperlipidemia	19 (12)
Normal ECG	78 (48)
Maximum Troponin ratio^a^	14 (5–34)
CMR diagnosis	
Myocardial infarction	40 (25)
Takotsubo syndrome	60 (27)
Normal	61 (37)
Impaired LV function	2 (1)

Values are presented as mean or median (range), or numbers (%)

*CAD* coronary artery disease, *CMR* cardiovascular magnetic resonance imaging, *ECG* electrocardiogram, *LV* left ventricular, *MINOCA* myocardial infarction with none-obstructive coronary arteries

^a^Ratio of maximum troponin divided by the upper limit of normal

### Comparison between CCTA and ICA

**Fig. 2:**
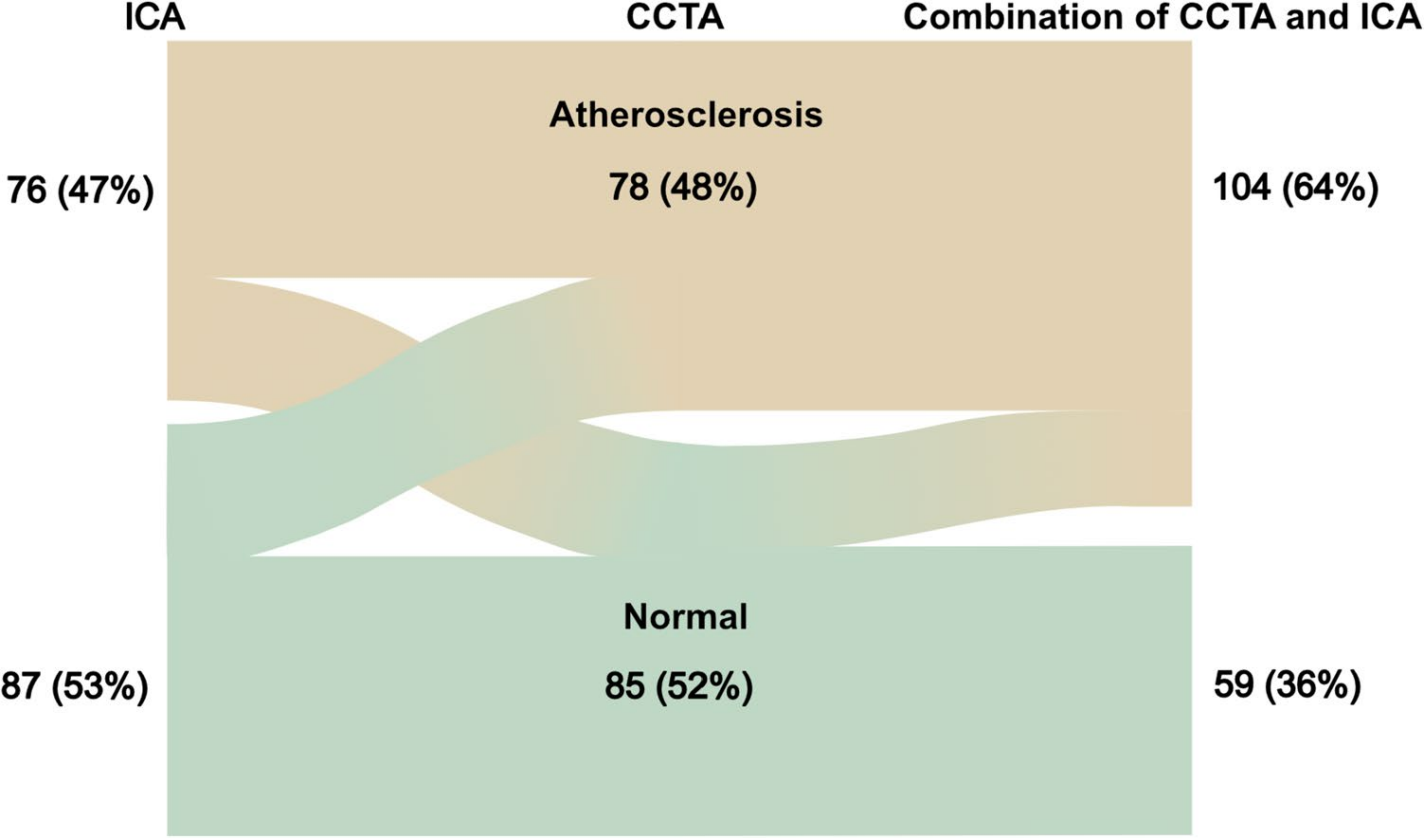
Coronary atherosclerotic segments were identified using ICA or CCTA separately, as well as in combination at the patient level, and are presented as numbers with corresponding percentages. *CCTA* coronary computed tomography angiography, *ICA* invasive coronary angiography

We studied the two methods’ respective ability to detect atherosclerosis in all assessable segments. CCTA detected atherosclerotic segments in 48% of the subjects whereas ICA detected atherosclerotic segments in 47% of the subjects. The discrepancy in interpretation, was not significant, according to McNemar’s test. Disagreement between CCTA and ICA was observed in 33% of the subjects when all segments were analyzed. The distributions of atherosclerotic segments, and the proportions with agreement, are presented in Fig. 1. CCTA and ICA agreed on completely normal coronary arteries in 36% of subjects, whereas they both detected atherosclerosis in any coronary artery segment in 31% (Table 2). The kappa value for agreement between CCTA and ICA was 0.34 (95% CI 0.19–0.48), with a significance level of *p* < *0.0001*. When CCTA was combined with ICA, significantly more patients with coronary atherosclerosis were found than with the baseline variable ICA alone (*p* < *0.001*). Figure 2 illustrates how the combination of CCTA and ICA significantly improved the detection of coronary atherosclerosis in MINOCA patients, increasing it from 47 to 64%.

**Table 2:** Poor agreement between CCTA and ICA

	CCTA	
ICA	Atherosclerosis	Normal
All segments		
Atherosclerosis	50 (31%)	26 (16%)
Normal	28 (17%)	59 (36%)
Proximal segments		
Atherosclerosis	45 (27%)	18 (11%)
Normal	29 (18%)	71 (44%)

Cross-tabulation of coronary atherosclerosis detected with CCTA and ICA in all and proximal comparable coronary segments at the patient level, presented as number of patients and proportions. Inter-method agreement kappa = 0.34 (95% CI 0.19–0.48) for all segments and 0.41 (95% CI 0.27–0.55) for proximal segments (both *p* < *0.0001)*

*CCTA* Cardiac computed tomography angiography, *ICA* Invasive coronary angiography

### Comparison between CCTA and ICA in proximal segments

Proximal segments (segments 1, 2, 5, 6, 7 and 11) were also compared, as the limited spatial resolution of CCTA may prevent the evaluation of distal segments less than 1.5 mm in diameter. The results of CCTA and ICA for proximal segments are shown in Table 2 and Supplemental Table 2. CCTA detected atherosclerotic segments in 45% of the subjects, whereas ICA found atherosclerotic segments in 39% of the subjects. The discrepancy in interpretation was not significant according to McNemer’s test. CCTA and ICA agreed that proximal segments were normal in 44% of the subjects, and that coronary atherosclerotic segments were present in 27% of patients. The inter-method agreement between CCTA and ICA regarding proximal segments, was 0.41 (95% CI 0.27–0.55, *p* < *0.0001*).

Table 2 shows that CCTA detected atherosclerotic segments that could not be seen with ICA in 18% of the subjects. The locations of the coronary atherosclerotic segments in these subjects are illustrated in Supplemental Fig. 1.

**Fig. 3:**
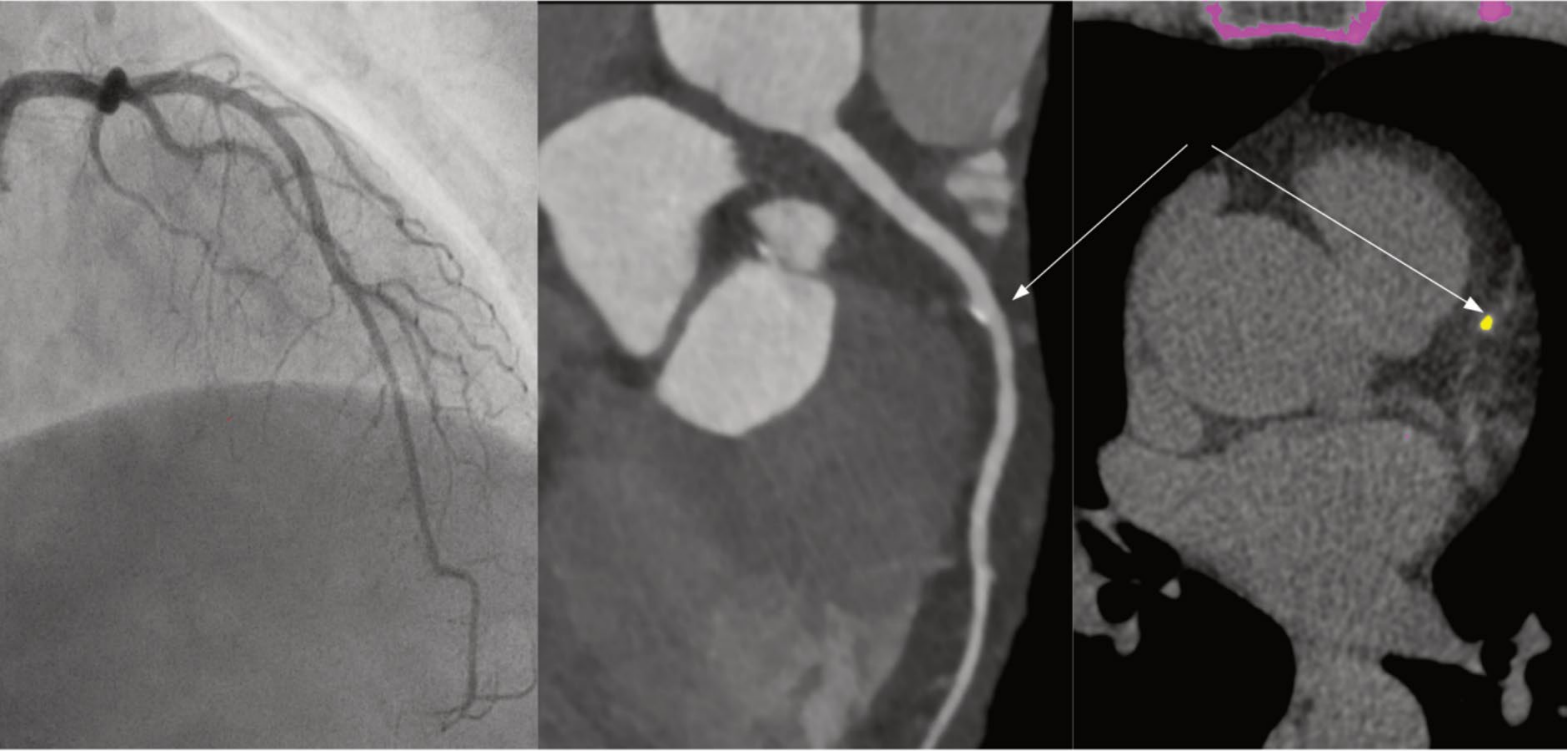
The left descending artery, with a calcified plaque in segment 6 evident only on CCTA (arrows). *CCTA* coronary computed tomography angiography

The combination of CCTA and ICA resulted in a statistically significant difference in detection rate compared with ICA alone, with CCTA having incremental value in detecting coronary atherosclerosis (*p* < *0.001*). Figure 3 illustrates a small, calcified plaque in the left descending artery detected with CCTA but not with ICA.

## Discussion

To our knowledge, SMINC-1 and SMINC-2 are the largest prospective studies of MINOCA patients where participants have been examined with CCTA and ICA in accordance with a predefined study protocol. The main findings of this sub-study are (1) that calcified, mixed, and non-calcified plaques are common in MINOCA patients, and (2) that the agreement between CCTA and ICA is poor, and (3) that CCTA adds information about atherosclerotic coronary segments. At a first glance, CCTA and ICA appeared to be equivalent in their ability to detect atherosclerotic coronary segments in MINOCA patients. However, agreement between the methods was limited. In only 31% of subjects, both methods detected atherosclerotic coronary segments in MINOCA patients, and in only 36% of subjects, both methods agreed that the coronary arteries were completely normal. As the spatial resolution of CCTA is inferior to that of ICA, we also performed analyses on proximal segments only. CCTA identified atherosclerotic coronary segments in 18% of subjects initially assessed as normal by ICA. One reason for the differences between CCTA and ICA in detection of coronary atherosclerosis is that ICA visualizes all intraluminal bulging of the intima and media only, excluding the adventitia, whereas CCTA visualizes all components of the artery wall, thus allowing detection of atherosclerotic plaques even when the coronary artery lumen is not affected. Another reason for the differences seen in atherosclerosis detection between CCTA and ICA is the limited spatial resolution of CCTA. This results in difficulties in assessing small peripheral segments with CCTA, meaning that pathology in such segments can be missed. The ability of CCTA to detect pathology in small artery segments, e. g., occlusion or dissection, may partly be related to individual factors, such as patient size and heart rate, and to technical parameters, such as the tube potential.

CCTA detected coronary atherosclerotic segments in 48% of MINOCA patients, and ICA in 47%, when all assessable segments were examined. In most subjects with coronary atherosclerosis, only a few segments were involved. Our findings, showing coronary atherosclerotic segments in 48% of patients undergoing ICA, are consistent with a recently published meta-analysis, which reported a prevalence of 53% [21]. Our CCTA findings also correspond with those of a recent Swedish population-based study that evaluated the prevalence of coronary atherosclerosis in healthy individuals using CCTA [22]. Other studies [10–12, 15] have shown similar results to this study, with the incidence and extent of atherosclerotic plaques seen with CCTA being relatively high in MINOCA patients, and often not detected with ICA. However, these studies were limited by the small numbers of patients included. Our study with a large sample size, shows that CCTA and ICA differ in their diagnostic ability to localize atherosclerotic plaques in the coronary arteries and that there is a lack of agreement between the methods in some coronary segments.

MINOCA patients have an increased risk of cardiovascular diseases such as reinfarction, stroke, and heart failure [23]. In a meta-analysis, the reported rate of reinfarction, stroke, and heart failure within 12 months was 9.6% [24]. The presence of atherosclerotic coronary segments seen with CCTA, including non-obstructive coronary artery disease (CAD), is associated with later clinical outcomes, including incident cardiac events [25–27], which highlights the importance of enhancing the detection of atherosclerotic coronary plaques in the MINOCA population.

In the large, multicenter SCOT-HEART trial, investigation with CCTA reduced the occurrence of death from coronary heart disease up to 5 years after CCTA [28]. This may be attributable to the use of targeted medical therapy, including initiation of, and adherence to, lipid-lowering agents in patients with coronary atherosclerotic plaques [29, 30].

Furthermore, the majority of MINOCA patients are female. Females with non-obstructive CAD on CCTA are at higher risk of major cardiovascular events compared to those with normal coronary arteries, according to a pooled analysis from the PROMISE and SCOT-HEART studies [31].

CCTA and ICA have complementary roles in detecting atherosclerosis in MINOCA, but the causative role of atherosclerosis remains unclear. Future research should aim to clarify the prognostic and clinical implications of non-obstructive atherosclerosis in MINOCA and determine whether CCTA can provide insights that influence patient management. Longitudinal studies, interventional trials, and advanced imaging techniques, such as pericoronary fat attenuation, may provide further insights. Based on the findings of this study, we propose that future research explore the role of CCTA after ICA in MINOCA patients to better characterize atherosclerosis and assess its clinical relevance.

### Strengths and limitations

The strengths are the relatively large sample size and that high quality CCTA and ICA images were reviewed by readers with many years of experience. The same readers also analyzed the images from both SMINC-1 and SMINC-2, blinded to all clinical information.

A limitation of this study is the timing of CCTA, which was performed after ICA, with an average delay of 3–6 months in SMINC-1 and 1 month in SMINC-2. Pathological findings detected on ICA, such as thrombotic lesions, may have resolved over time, and pharmacological treatment including statins could have influenced the composition and extent of coronary atherosclerosis before CCTA was conducted. Plaques were visually assessed and categorized as non-calcified, partially calcified, or calcified, which does not provide sufficient information to determine high-risk plaque characteristics. Pericoronary fat attenuation was not measured at the time of CCTA analysis, although this method has recently been shown to provide prognostic information in patients with coronary artery disease [32]. The inclusion criteria differed slightly between SMINC-1 and SMINC-2, which may have influenced the assessment of coronary atherosclerosis. In SMINC-1, patients with coronary artery plaques exceeding 30% stenosis were excluded, whereas in SMINC-2, the threshold was 50%, aligning with the current MINOCA definition. This discrepancy may have led to an underestimation of the atherosclerotic burden in the SMINC-1 cohort and impacted the comparison between CCTA and ICA, as CCTA is more sensitive in detecting small plaques. The inclusion of TTS patients may have contributed to the high proportion of female patients and, in turn, have influenced the observed prevalence of atherosclerosis. Our study is also partly limited by the age criteria for participants. The exclusion of patients under 35 may have led to a lower prevalence of specific MINOCA causes, such as spontaneous coronary dissection (SCAD) and vasospasm, compared to the general MINOCA population. Similarly, excluding patients over 70, who typically have a higher atherosclerotic plaque burden, may reduce the generalizability of our findings. In this study, we did not report the prevalence of SCAD. Since CCTA was performed some time after ICA, any SCAD had likely already healed, making a direct comparison between ICA and CCTA for SCAD diagnosis challenging. Other limitations of this analysis include the fact that only visual assessment of the coronary arteries was performed. No intracoronary imaging, or invasive or non-invasive testing for microvascular dysfunction or vasospasm, was performed. Additionally, no long-term follow-up of patients with coronary plaques was conducted. Some degree of subjectivity is also inherent to radiologic interpretation and bias therefore cannot be ruled out entirely.

## Conclusions

MINOCA patients frequently exhibit coronary plaques. Although CCTA and ICA detect similar proportions of pathology overall, they demonstrate poor agreement in MINOCA cases. As anticipated, CCTA identified plaques that were not detected by ICA. Surprisingly, CCTA also failed to detect a significant number of plaques identified by ICA. CCTA provides substantial and relevant information about coronary atherosclerotic segments, offering valuable additional diagnostic insights into plaque burden. Thus, CCTA should be regarded as a complementary tool to ICA, potentially enhancing risk stratification and guiding medical therapy in the context of MINOCA.

## Supplementary Information

Below is the link to the electronic supplementary material.

Supplementary file1 (DOCX 372 kb)

## Data Availability

No datasets were generated or analysed during the current study.

## Abbreviations

CAD: Coronary artery disease
CCTA: Coronary computed tomography angiography
CMR: Cardiovascular magnetic resonance imaging
ECG: Electrocardiogram
ICA: Invasive coronary angiography
LV: Left ventricular
MI: Myocardial infarction
MINOCA: Myocardial infarction with nonobstructive coronary arteries
SCAD: Spontaneous coronary artery dissection
