# Supplementary material for: Incremental value of coronary computed tomography angiography in addition to invasive coronary angiography in MINOCA
Source: Int J Cardiovasc Imaging. 2025 Apr 21;41(6):1151–60. doi: 10.1007/s10554-025-03401-1 (PMC12162781; doi:10.1007/s10554-025-03401-1)
Supplement: Supplementary file 1 — Supplementary file1 (DOCX 372 kb) [file 10554_2025_3401_MOESM1_ESM.docx]

**Supplemental Materials**

CCTA data acquisition

Examinations were performed on one of two multidetector CT scanners:

1. A 64 row CT scanner (LightSpeed VCT XT; GE Healthcare, Milwaukee, Wisconsin, USA) using a prospectively ECG-triggered scan protocol: detector configuration 64 x 0.625 mm, rotation time 350 ms, tube potential 120 kV, tube current 450–650 mAs (depending on patient size). The contrast agent used was iodixanol 320 mg I/ml (Visipaque, GE Healthcare, Stockholm, Sweden), which was administered using a triple-phase protocol and individually dosed, based on body weight (400 mg I/kg, 75–100 ml iodixanol), with a fixed injection time (15 s).
2. A dual-source 2x64 row CT scanner with a Stellar detector (Somatom Definition Flash, Siemens Medical Solutions, Forchheim, Germany) using a prospectively ECG-triggered scan protocol: detector configuration 128 x 0.6 mm, rotation time 280 ms, tube potential 100–120 kV, tube current 320–340 mAs (depending on patient size). The contrast agent used was iodixanol 320 mg I/ml (Visipaque, GE Healthcare, Stockholm, Sweden), with a dose of 325 mg I/kg and a fixed injection time (12 s).

In the absence of contraindications, patients received oral metoprolol (25–100 mg, depending on initial heart rate) 1 hour prior to the examination. In addition, patients with a systolic blood pressure >110 mmHg received sublingual nitroglycerine (0.4 mg) 4 minutes before the scan.

**Supplemental Figure 1. Atherosclerotic segments seen only with CCTA**

Distribution of proximal atherosclerotic coronary segments seen with CCTA but not with ICA (number of segments from 29 subjects). The segment numbers are written in white digits.


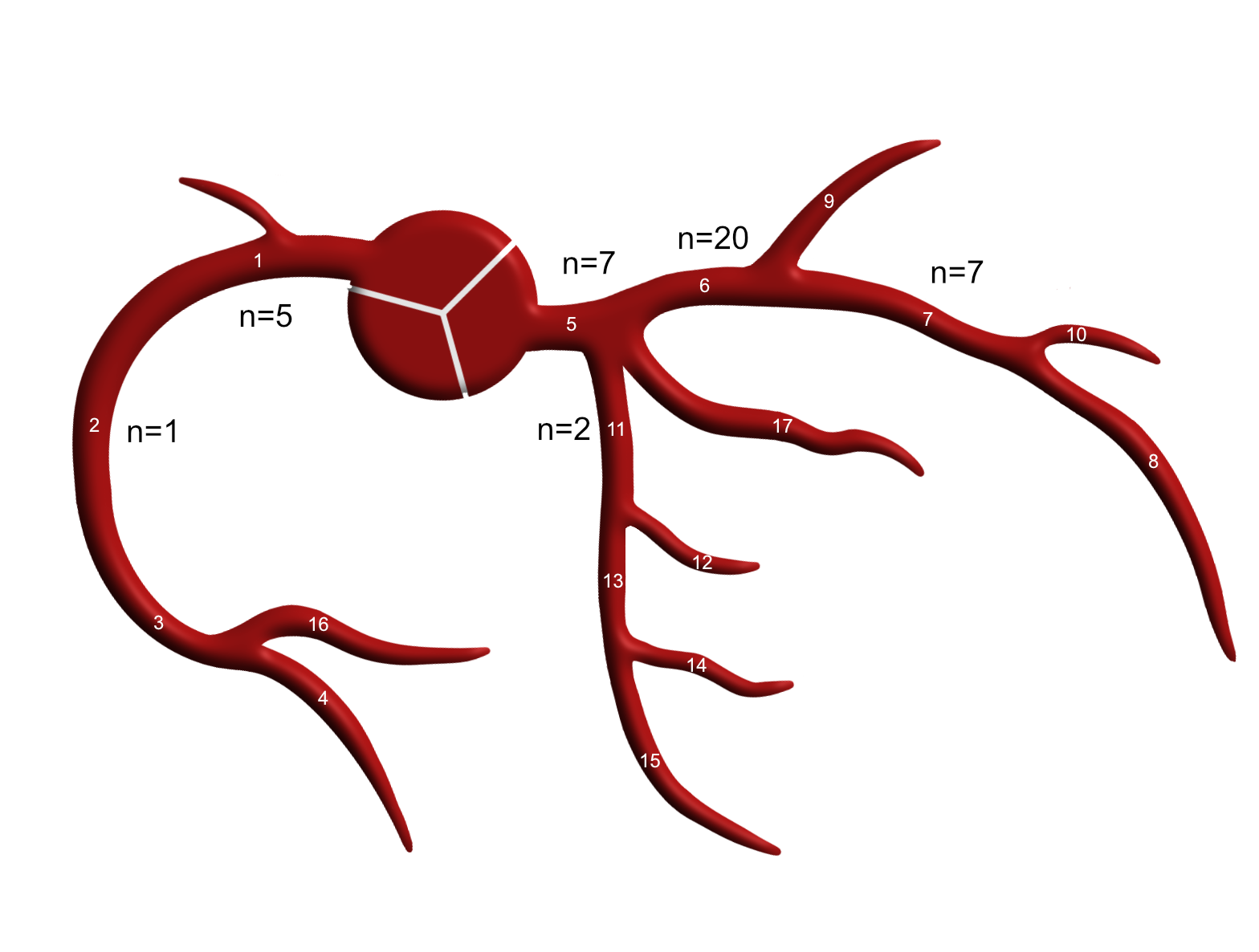


Abbreviations: CCTA, coronary computed tomography angiography; ICA, invasive coronary angiography.

**Supplemental Table 1.** CCTA plaque burden and composition.

|  |  | | MINOCA patients | | |
| --- | --- | --- | --- | --- | --- |
|  |  | | n=163 | CAC^a^ | |
| **All CAD** | 0 segments | | 85 (52%) | 0 | |
|  | 1 segment | | 39 (24%) | 32 (0–778) | |
|  | 2 segments | | 11 (7%) | 36 (0–120) | |
|  | 3 segments | | 9 (6%) | 86 (5–240) | |
|  | 4 segments | | 8 (5%) | 52 (0–146) | |
|  | 5 segments | | 4 (2%) | 41 (5–108) | |
|  | 6 segments | | 1 (1%) | 169 | |
|  | 7 segments | | 2 (1%) | 604 (382–828) | |
|  | 8 segments | | 1 (1%) | 292 | |
|  | 9 segments | | 3 (2%) | 567 (123–1,176) | |
|  | 10 segments | | 0 | -- | |
|  | | |  | | |
| **Plaque composition** | All n=163 | No CAD | | | 85 (52%) |
|  |  | Any non-calcified plaque | | | 13 (8%) |
|  |  | Mixed plaque | | | 21 (13%) |
|  |  | Calcified plaque | | | 64 (39%) |
|  | Females n=121 | No CAD | | | 65 (54%) |
|  |  | Any non-calcified plaque | | | 7 (6%) |
|  |  | Mixed plaque | | | 14 (12%) |
|  |  | Calcified plaque | | | 48 (40%) |
|  | Males n=42 | No CAD | | | 20 (48%) |
|  |  | Any non-calcified plaque | | | 6 (14%) |
|  |  | Mixed plaque | | | 7 (17%) |
|  |  | Calcified plaque | | | 16 (38%) |
| Abbreviations: CAC, coronary artery calcium score (Agatston Units); CAD, coronary artery disease; CCTA, coronary computed tomography angiography; MINOCA, myocardial infarction with none-obstructive coronary arteries. Values are presented as absolute values (percentage) or median (range). ^a^Median CAC calculated in participants with CAC score >0. | | | | | |

**Supplemental Table 2.** All inter-method agreement Cohen's kappa values (k) regarding proximal segments and by CMR diagnosis.

| **CMR diagnosis** | |  | **Cohen’s kappa values** |
| --- | --- | --- | --- |
|  | All subjects (n=163) |  | 0.41 (95% CI 0.27–0.55), *p <0.0001* |
|  | Myocardial infarction (n=40) |  | 0.36 (95% CI 0.07–0.66), *p =0.021* |
|  | Takotsubo syndrome (n=60) |  | 0.60 (95% CI 0.40-0.80), *p <0.0001* |
|  | Normal CMR (n=61) |  | 0.27 (95% CI 0.03-0.51), *p =0.031* |
| Abbreviations: CMR, cardiovascular magnetic resonance imaging | | | |
